# Clinical and Microbiological Effects of an Antimicrobial Stewardship Program in Urology—A Single Center Before-After Study

**DOI:** 10.3390/antibiotics11030372

**Published:** 2022-03-10

**Authors:** Oana Joean, Daniel Tahedl, Madita Flintrop, Thorben Winkler, Ruxandra Sabau, Tobias Welte, Markus A. Kuczyk, Ralf-Peter Vonberg, Jessica Rademacher

**Affiliations:** 1 Department of Respiratory Medicine, Hannover Medical School, 1st Carl-Neuberg Street, 30625 Hannover, Germany; welte.tobias@mh-hannover.de (T.W.); rademacher.jessica@mh-hannover.de (J.R.); 2 Department of Urology and Urologic Oncology, Hannover Medical School, 1st Carl-Neuberg Street, 30625 Hannover, Germany; daniel.tahedl@posteo.de (D.T.); flintrop.madita@mh-hannover.de (M.F.); winkler.thorben@mh-hannover.de (T.W.); kuczyk.markus@mh-hannover.de (M.A.K.); 3 Institute for Clinical Pharmacology, Hannover Medical School, 1st Carl-Neuberg Street, 30625 Hannover, Germany; sabau.ruxandra@mh-hannover.de; 4 Biomedical Research in Endstage and Obstructive Lung Disease (BREATH), German Center for Lung Research (DZL), 1st Carl-Neuberg Street, 30625 Hannover, Germany; 5 Institute for Medical Microbiology and Hospital Epidemiology, Hannover Medical School, 1st Carl-Neuberg Street, 30625 Hannover, Germany; vonberg.ralf@mh-hannover.de

**Keywords:** antibiotics, antimicrobial stewardship, antimicrobial resistance, urology, quinolone

## Abstract

Antimicrobial resistance is a major public health issue caused by antibiotic overuse and misuse. Antimicrobial stewardship (AMS) has been increasingly endorsed worldwide, but its effect has been studied scarcely in urologic settings. A before-after study was performed from 2018 through 2020 to evaluate changes in antimicrobial prescription, resistance rates and clinical safety upon implementation of an AMS audit and feedback program in the Urology Department of a large German academic medical center. The primary endpoints were safety clinical outcomes: the rate of infection-related readmissions and of infectious complications after transrectal prostate biopsies. Resistance rates and antimicrobial consumption rates were the secondary endpoints. The AMS team reviewed 196 cases (12% of all admitted in the department). The overall antibiotic use dropped by 18.7%. Quinolone prescriptions sank by 78.8% (*p* = 0.02) and 69.8% (*p* > 0.05) for ciprofloxacin and levofloxacin, respectively. The resistance rate of *E. coli* isolates declined against ceftriaxone (−9%), ceftazidime (−12%) and quinolones (−25%) in the AMS period. No significant increase in infection-related readmissions or infectious complications after prostate biopsies was observed (*p* = 0.42). Due to the potential to reduce antibiotic use and resistance rates with no surge of infection-related complications, AMS programs should be widely implemented in urologic departments.

## 1. Introduction

Antimicrobial resistance is an urgent global public health threat brought about by these drugs’ overuse and misuse [1,2]. While they underpin all areas of modern medicine, antimicrobials’ inappropriate prescription and the subsequent resistance development have the potential to cause up to 10 million deaths by 2050 and to dramatically affect health-care systems and economies worldwide [3,4]. Even more alarming, recent data suggests that the toll of worldwide resistance-associated deaths might have been as high as 5 million in 2019 and that antibiotic consumption rates have continuously increased in the last two decades [5,6]. Moreover, due to rapid environmental dissemination, antimicrobial resistance threatens to become a planetary health issue [7]. In response to this imminent danger, antimicrobial stewardship (AMS) programs were endorsed across the globe with the aim to achieve optimal clinical outcomes with minimal unintended antimicrobial effects like toxicities, selection and spread of resistant pathogens [8]. While considerably variable worldwide, AMS programs include a set of actions that promote a responsible antimicrobials’ use at different public health levels [9].

Urinary tract infections are among the most common infections in both the community and in hospital settings [10]. Moreover, urologic patients are prone to developing hospital-acquired urinary tract infections, potentially with multi-drug-resistant pathogens [11]. A global surveillance study on urologic infections recruiting patients from over 70 countries showed that over half of hospitalized urologic patients received antibiotics, on whom in 46% of cases broad-spectrum antimicrobials were used for prophylactic purposes [12]. Furthermore, high global resistance rates for uropathogens were found, and these correlated with antibiotic consumption [13,14]. In line with that, fluoroquinolones, which have been some of the most commonly used antibiotics in urology for many years, were recently shown to promote resistance not only in patients treated with antibiotics, but also in their household contacts [15].

Despite the obvious need for rapid action, studies describing AMS interventions in urology are scarce. Additionally, outcomes in the available literature have focused on a decrease in antibiotic consumption and cost minimization through implementing an AMS program rather than on safety clinical measurements like the sepsis rate or infection-related readmission [16,17,18,19,20]. Our aim was to evaluate whether an AMS multifaceted care bundle including site-specific antimicrobial guidelines and weekly interdisciplinary rounds with infectious disease specialists and pharmacists could reduce antimicrobial consumption with no negative clinical effects.

## 2. Results

### 2.1. Patient Characteristics

During the pre-AMS period, 1556 patients were hospitalized in the Urology Department, corresponding to 7242 patient days, and 75 patients had a transrectal prostate biopsy performed in the outpatient clinic. Conversely, in the intervention period, 1636 patients were admitted for a total of 9015 patient days, and there were 76 prostate biopsy patients. There were no significant differences with respect to the primary diagnosis and hospital stay length, but the patients in the AMS period were slightly older (64 years (50–74) in the pre-AMS interval vs. 65 years (53–75) in the AMS period, *p* = 0.46) (Table 1). The case-mix indexes calculated for the two cohorts did not significantly differ (1.08 (1.06; 1.11) in the pre-AMS period vs. 0.97 (0.96; 1.03) in the AMS period, *p* = 0.11).

### 2.2. Antimicrobial Stewardship Implementation

**Figure 1:**
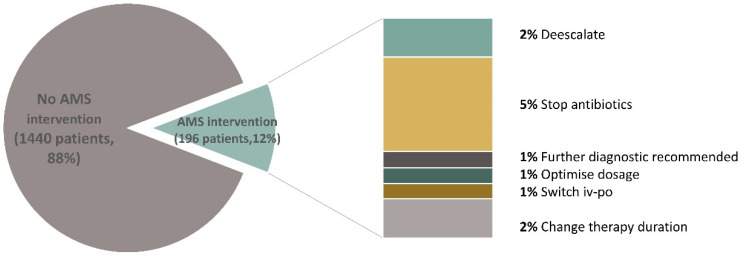
Antimicrobial stewardship interventions. AMS: antimicrobial stewardship.

Due to restricted non-essential patient contact during the high incidence timespans of the COVID-19 pandemic, only 38 weekly interdisciplinary rounds took place in the year 2020. During the rounds, 196 patients (12% of all hospitalized) were discussed with the bedside physicians. The AMS team recommended they de-escalate in 18% of them, stop antibiotic treatment in 42%, perform further diagnostic procedures (e.g., additional cultures or radiology) in 7%, and a dosage optimization in 8% according to drug trough levels, to the kidney function or to the patients’ weight. In 7% of the discussed cases, a switch from the intravenous to the oral application route was suggested, and in 18% of the discussed cases the therapy duration was recommended (Figure 1). Adherence to the recommendations was observed in 89% of cases.

### 2.3. Antimicrobial Consumption

**Figure 2:**
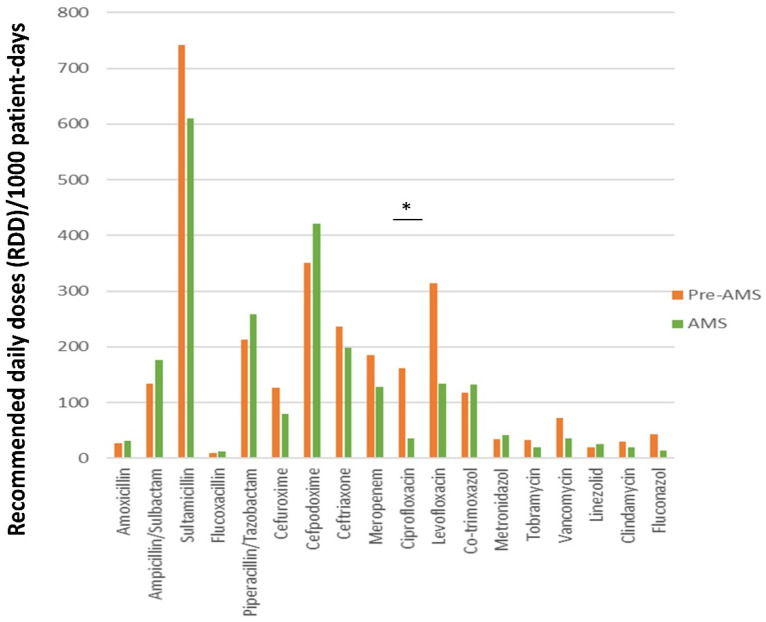
Changes in antimicrobial prescription patterns during the antimicrobial stewardship program; green bars: pre-antimicrobial stewardship; yellow bars: antimicrobial stewardship; * *p* < 0.05, AMS: antimicrobial stewardship.

In the AMS intervention year, the overall antibiotic use dropped by 18.7% (543.2 recommended daily doses/1000 patient days, RDD/1000 patient days). Fluoroquinolone prescriptions plummeted by 78.8% (134 RDD/1000 patient days, *p* = 0.02) and 69.8% (310 RDD/1000 patient days, *p* > 0.05) for ciprofloxacin and levofloxacin, respectively (Figure 2). This phenomenon was, however, coupled by a rise in the use of ampicillin/sulbactam and cefpodoxime (by 30% and 39%, respectively) that replaced the quinolones as a perioperative prophylaxis and also by an increased piperacillin/tazobactam utilization (by 24%, 50 RDD/1000 patient days). Moreover, the broad-spectrum antibiotics meropenem and vancomycin were used less frequently and therefore showed a drop in RDDs by 22.4% and 55.4%, respectively (Figure 2). Lastly, the fluconazole consumption was reduced by 765.8%, and ceftriaxone was used 21.9% less than in the previous period (Table S1).

### 2.4. Microbiological Results

**Figure 3:**
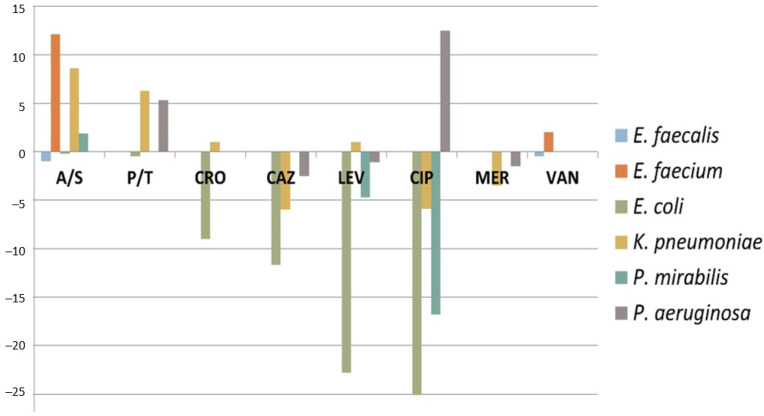
The dynamics of the antimicrobial resistance patterns for *E. faecalis*, *E. faecium*, *E. coli*, *K. pneumoniae*, *P. mirabilis* and *P. aeruginosa* upon implementation of an antimicrobial stewardship program in the urology department. A/S: Ampicillin/Sulbactam; P/T: Piperacilin/Tazobactam; CRO: Ceftriaxone; CAZ: Ceftazidime; LEV: Levofloxacine; CIP: Ciprofloxacine; MER: Meropenem; VAN: Vancomycin.

During the intervention, we observed no significant change in the number of analyzed blood cultures and urinary samples (357 vs. 337 blood cultures and 1323 vs. 1318 urinary specimens). The resistance rate of *E. coli* isolates against ceftriaxone, ceftazidime and quinolones declined in the AMS period. This phenomenon was most prominent for levofloxacin and ciprofloxacin, with a drop of almost 25% (from 26% to 18.6% for ciprofloxacin). In contrast, we observed slight increases (10%) in the ampicillin/sulbactam resistance rates of *E. faecium* and *K. pneumoniae* isolates and a minimal raise (5%) in the piperacillin/tazobactam resistance rates of *K. pneumoniae* and *P. aeruginosa* isolates (Figure 3). Surprisingly at first glance, there were around 12% more *P. aeruginosa* isolates resistant to ciprofloxacin in the AMS period, despite a dramatic drop in prescriptions (Figure 3). When revising the medical charts in more detail, however, it became clear that these patients had acquired the infections outside the hospital.

In order to assess whether the decreased resistance rate of *E. coli* against quinolones was a reflection of a global trend due to restricted drug use after various warnings regarding adverse effects, we compared the data from the Urology department to overall data from all urinary samples analyzed in the hospital. The overall *E. coli* resistance rate against ciprofloxacin was 22% in 2020 and 21% in 2021.

### 2.5. Infectious Complications

Although hospital readmission was more frequent for the pre-AMS cohort (362 patients (23%) vs. 176 patients (10.5%), *p* = 0.0001), no statistical difference was observed with regard to infection-related readmissions (31 patients (2%) vs. 22 patients (1.3%), *p* = 0.42). In terms of infectious complications after a transrectal prostate biopsy, only one patient (1/77) experienced fever and required admission in the AMS period, while no infectious complication (0/76) occurred in the previous period (Table 1).

## 3. Discussion

Our results show that an AMS intervention including site-specific antimicrobial guidelines, a weekly audit and feedback rounds with infectious disease specialists and pharmacists led to a reduction in the overall antibiotic use, to a significant drop in quinolone prescriptions and to a consecutive decrease in quinolone resistance rates with no surge of infection-related complications. Moreover, after one AMS year, the *E. coli* resistance rate against ciprofloxacin in urologic patients was lower than the overall hospital rate, which had not shown any changes in the analyzed period.

However, we also observed a slight increase in ß-lactam resistance rates upon switching to ß-lactam-based prophylaxis regimens. The study underscores the causality between antimicrobial use and resistance emergence, which demands continuous evaluation and advocates for the safety of antimicrobial stewardship programs in urology departments. Due to substantial antibiotic consumption in these divisions, a widespread implementation of AMS programs tailored for urologic pathologies has the potential to impact resistance patterns for whole communities. While we acknowledge that patients in urology departments oftentimes require antimicrobial therapies, clinical practices should be continuously optimized based on scientific progress.

The optimal anti-infective management of urinary tract infections implies the following criteria: selecting the correct drug and dose and the shortest clinical effective duration of therapy. Antibiotics should be chosen based on the local resistance rate, no empiric fluoroquinolone prescriptions and no antibiotic prescriptions for asymptomatic bacteriuria as well as on the urinary culture before starting an antibiotic therapy [21,22]. Lamentably, the literature on the implementation of an AMS program in urology is scant, and the few available studies were performed only in Europe or North America, thus missing the chance to deal with an overarching global problem [16,17,18,23,24,25].

A recent randomized controlled trial could show that omitting antimicrobial prophylaxis in patients having a transurethral prostrate resection without preoperative pyuria and/or an indwelling catheter was safe, thus challenging both the European and the American Urology Associations’ guidelines [26]. Similar results have been published for antibiotic prophylaxis after urethroplasty, showing no difference in stricture recurrences or wound complications between patients who had a negative or a positive culture and thus suggesting that postoperative prophylaxis may in fact offer no benefit [27]. In an observational retrospective study that included adults with a urinary tract infection with ESBL-producing *E. coli*, an AMS program significantly increased the cure rate [24]. Furthermore, an AMS intervention performed over five years in Japan achieved a decrease in inappropriate broad-spectrum antibiotic use paralleled by a rise in the rate of microbiological sampling [28]. Dik et al. studied an AMS intervention cohort for antibiotic treatments in urologic wards (114 intervention and 357 control cases) and showed a significant drop in antimicrobial consumption for all patients and a decreased length of hospital stay [17]. The successful results from the abovementioned AMS urology interventions are in line with our results. To the best of our knowledge, no other AMS intervention performed in urology was shown to lower resistance rates.

The fact that AMS should not be interrupted has been underlined by the results of Jang and colleagues, who showed that by discontinuing an AMS program the old antimicrobial use patterns reappeared [29]. Although fewer AMS rounds took place than initially planned and despite the fact that we only directly discussed 12% of the hospitalized patients, the program could still affect change through the other pillars of the intervention: hospital-specific guidelines and educational sessions. The acceptance rate of 88% to the AMS team’s recommendations was higher than in previous studies, in which it varied from 41% to 86.8% [24,25]. This undoubtedly contributed to the intervention’s effects and was eased by the strong interest in an adequate antimicrobial therapy shown by the members of the urologic team. We believe that bedside clinicians should be involved in every step of a successful antimicrobial stewardship program in order to achieve optimal results that are in patients’ best interests: an efficient infection treatment and the avoidance of unnecessary and potentially dangerous overtherapy.

The higher prescriptions of piperacillin/tazobactam were at least partly caused by the fact that this drug was now the first-line therapy for infections with *P. aeruginosa* and that an increased dose of 4/0.5 g four times daily was preferred in the AMS period in contrast to the historic cohort. The increased dosage was based on the current recommendations of the European Committee on Antimicrobial Susceptibility Testing (EUCAST) [30].

Our study’s major limitation is the comparison to a historical cohort. We chose not to randomize the patients because our aim was to design a pragmatic intervention that reflected standard care. Moreover, we aimed to avoid the potential confounder due to changes in physicians’ prescription practices during the intervention. We also did not stratify the patients by pathologies or comorbidities (e.g., immunosuppression, indwelling catheter, previous antibiotic exposure, infection as a primary diagnosis vs. hospital-acquired), and therefore some of the results might have been averaged out. However, we sought to show population-wide effects and to advocate for a wide implementation of antibiotic stewardship programs and not only for some subpopulations. 

Due to its single-center nature, our study’s results might only be reproducible in the setting of a tertiary-level urology department and might be less reflective of smaller settings. An ideal study design would have been a multi-center cluster randomized study comparing an AMS intervention to the standard of care in centers from different geographical regions and of different sizes. However, the barriers to implementing AMS in surgical departments are, at least in our experience, high. Therefore, recruiting enough centers to attain statistical power for such a study would have been an utmostly difficult task. We hope, however, that our data will provide an incentive to perform further larger studies.

Unfortunately, we did not have any data on resistance rate trends in the outpatient setting, so we cannot estimate to what degree our data were a reflection of prescription patterns outside of the hospital.

## 4. Materials and Methods

### 4.1. Study Design

A quasi-experimental, before-after study [31] was performed to evaluate changes in antimicrobial prescription behavior, antimicrobial resistance rates and clinical safety upon implementation of an antimicrobial stewardship audit and feedback program in the Urology Department of a large academic medical center.

### 4.2. Hospital Setting and Implementation of an Antimicrobial Stewardship Program

**Figure 4:**
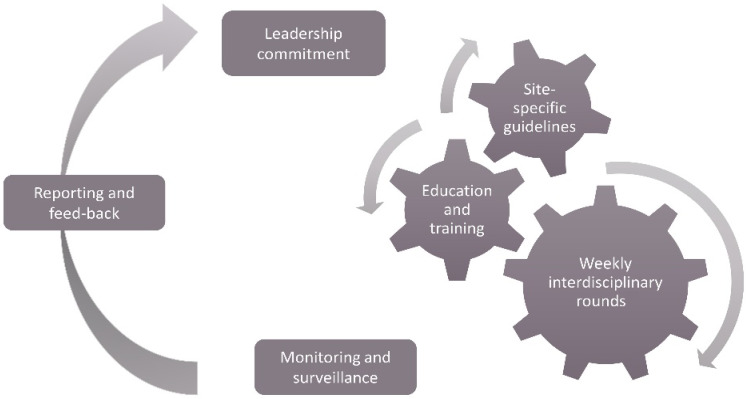
The antimicrobial stewardship intervention in the urology department.

Hannover Medical School is a 1520-bed maximum care academic hospital with a nationwide catchment area, and approximately 2000 patients are treated yearly in the Urology Department. An AMS intervention was implemented in the Urology Department from 1 January through 31 December 2020 and involved a bundle-of-care consisting of site-specific guidelines, education and training as well as weekly interdisciplinary rounds. Urologists, infectious disease physicians, microbiologists and pharmacists developed site-specific antimicrobial guidelines for perioperative antibiotic prophylaxis and the management of urologic infections based on local antimicrobial resistance patterns and on the national and international recommendations (Table S2) [26,32,33,34]. The guidelines additionally contained preferred and alternative empiric antimicrobial choices as well as duration of therapy recommendations. They also include recommendations on adjusting the therapy based on microbiological results and describe specific perioperative regimens tailored for every type of surgical intervention. Due to increasing resistance rates and to their potential for long-lasting adverse effects, quinolones were not recommended for empiric treatments and were excluded from the perioperative prophylaxis regimens in favor of ampicillin/sulbactam. The rollout of the new guidelines was accompanied by educational and training sessions for urologists. The weekly rounds were performed by an interdisciplinary team (clinical microbiologist, infectious diseases specialist, and pharmacologist) that discussed the current therapies with the bedside physician (Figure 4). The antibiotic regimens and the available diagnostics were reviewed, and recommendations on the continuation, medication switch, additional diagnosis, dosage adjustment or drug trough-level measurements were made. However, the urologists maintained prescribing autonomy. The AMS team also offered support between the standard rounds if necessary. Data on antibiotic prescriptions and resistance patterns were monitored by the hospital’s pharmacy and microbiology department, respectively, and were reported on a regular basis to each clinical department.

### 4.3. Study Population and Historic Cohort

All hospitalized adult urologic patients and all patients who had a transrectal prostate biopsy in an outpatient setting between January and December 2020 were included in the AMS cohort, and the follow-up time was three months (until March 2021). In order to evaluate the clinical, microbiological and performance effects of the AMS intervention, a historic control cohort was compiled by including the same group of patients treated between October 2018 and September 2019 in the urology department. The washout period of three months between the cohorts (October–December 2019) was chosen to allow for an adequate follow-up of patients in the historic cohort and for an AMS rollout including educational and training sessions for urologists.

We tried to avoid a selection bias by including all patients in the abovementioned time intervals. In order to adjust for eventual clinical and structural changes due to the COVID-19 pandemic that was coincident with the AMS cohort, we included the case-mix index as a dependent variable. The case-mix index is the relative value allocated to the diagnosis-related group of hospitalized patients and reflects the complexity, diversity and needs for resources in the analyzed populations.

### 4.4. Data Collection

We collected clinical demographic data (age, primary diagnosis and the corresponding diagnosis-related group (DRG), case-mix index, admission and discharge date, all-cause and infection-related readmission date(s), if applicable, infectious complications after a transrectal prostate biopsy) from an institutional patient data interface (SAP GUI for Windows, SAP SE). Data regarding antibiotic treatment, type of infection, recommendations from the AMS team and acceptance rate for the recommendations were collected in an institutional patient database (FileMaker Pro™, Claris International Inc., Santa Clara, CA, USA). Furthermore, microbiological information (number of performed microbiological diagnostics from urinary samples and blood cultures, diagnostic rate for urinary pathogens and resistance rate) was retrieved from the internal server of the Microbiology Department. All data were collected prospectively, and the analysis was performed retrospectively between March and July 2021.

### 4.5. Primary and Secondary Endpoints

The primary endpoints were safety clinical outcome measures: the rate of infection-related readmissions and the rate of infectious complications after transrectal prostate biopsies. As secondary endpoints, we chose microbiological and performance outcome measures. The microbiological endpoints were the rate of uropathogens resistant to the most commonly used antibiotics: ampicillin/sulbactam, piperacillin/tazobactam, ceftrazidime, ceftriaxone, ciprofloxacin, levofloxacin, meropenem and vancomycin. The performance endpoints were measures of antibiotic use (recommended daily doses (RDD)/1000 patient days, as defined by the World Health Organization [35]).

### 4.6. Statistical Analysis

The data were analyzed using the IBM SPSS Statistics (version 26.0, IBM Corp., Armonk, NY, USA) program.

The assumption of normality was tested for continuous variables using the Shapiro-Wilk normality test. Continuous variables are summarized as medians and with the first and third quartile (Q1 and Q3), whereas categorical variables are presented as absolute numbers (*n*) or percentages (%). Continuous variables were compared by a Mann-Whitney U-test. For categorical variables, the chi-square test or Fisher’s exact test was used as appropriate. A two-tailed *p*-value of ≤0.05 was considered statistically significant. 

### 4.7. Ethics Approval and Consent to Participate

All patients provided consent upon admission to the hospital to use data acquired during the clinical routine for research purposes. The study was conducted in accordance with the ethical guidelines of the 1975 Declaration of Helsinki and was approved by the internal review board (9670_BO_K_2021).

## 5. Conclusions

Antimicrobial resistance is an imminent public health threat that requires urgent action and is a challenge to urologic practice. AMS programs carried out by infectious disease specialists, pharmacologists and motivated urologic surgeons should be implemented widely in order to improve patient outcomes and fight against this global health issue.

## Figures and Tables

**Table 1:** Patients‘ characteristics.

Variable	Pre-AMS *	AMS	*p*-Value
Total number of patients	1.556	1.636	
Patient age (median, IQR)	64 (50–74)	65 (53–75)	0.046
Primary diagnosis (*n*, %)			>0.05
Infectious	212 (13.6)	242 (14.8)	
Benign prostate hyperplasy	107 (6.9)	163 (10)	
Benign tumor	17 (1)	23 (1.4)	
Malignant tumor	529 (34)	709 (43.3)	
Obstructive uropathy	221 (14.2)	211 (12.8)	
Haematuria	38 (2.4)	-	
Lithiasis	205 (13.2)	283 (17.3)	
Other	226 (14.5)	229 (14)	
Days of hospitalization (median, IQR **)	3 (2–6)	3 (2–6)	>0.05
Readmissions	857	547	
Readmissions due to infections (*n*, % of total readmissions)	31 (3.6)	22 (4)	>0.05
Days until readmissions due to infections (median, IQR)	5 (2.5–9)	6.5 (4–10)	>0.05

* AMS: antimicrobial stewardship; ** IQR: interquartile range.

## Data Availability

Data will be made available by the corresponding author upon reasonable request. The data are not publicly available due to the European General Data Protection Regulation (GDPR).

## Supplementary Materials

The following supporting information can be downloaded at: https://www.mdpi.com/article/10.3390/antibiotics11030372/s1, Table S1: Recommendations for perioperative prophylaxis; Table S2: Antibiotic consumption in recommended daily doses.
