# Supplementary material for: Clinical and Microbiological Effects of an Antimicrobial Stewardship Program in Urology—A Single Center Before-After Study
Source: Antibiotics (Basel). 2022 Mar 10;11(3):372. doi: 10.3390/antibiotics11030372 (PMC8944612; doi:10.3390/antibiotics11030372)
Supplement: Supplementary file 1 [file antibiotics-11-00372-s001.zip › antibiotics-1593265-supplementary.pdf]

Table S1. Antibiotic consumption in recommended daily doses/ 1000 patient-days.

	Pre-AMS				AMS					
**Antibiotic**	**Q4 2018**	**Q1 2019**	**Q2 2019**	**Q3 2019**	**Q1 2020**	**Q2 2020**	**Q3 2020**	**Q4 2020**	**Difference (Absolute Values)**	**Difference (%)**
Amoxicillin	0	0,0	0,0	14,40	6,78	7,31	0,00	17,35	17,04	118,33
Ampicillin/Sulbactam	26,47	29,49	29,51	50,03	49,26	37,53	40,80	48,62	40,71	30,04
Sultamicillin	178,57	150,79	185,49	188,30	175,75	174,89	115,59	143,30	-93,62	-13,31
Flucloxacillin	0,74	0,00	1,83	0,00	5,09	7,58	0,00	0,00	10,1	393,00
Piperacillin/Tazobactam	51,76	59,02	55,37	42,39	56,04	64,12	75,57	62,53	49,72	23,84
Cefuroxime	38,15	42,13	21,43	24,27	6,78	9,18	35,36	27,62	-47,04	-37,34
Cefpodoxime	46,77	67,02	76,14	111,96	94,24	96,15	117,86	112,04	118,4	39,22
Ceftriaxone	96,37	62,23	38,76	55,98	81,51	38,46	36,26	41,69	-55,42	-21,88
Meropenem	42,52	42,27	30,45	49,21	30,57	24,73	27,92	44,29	-36,94	-22,46
Ciprofloxacin	62,36	52,66	24,92	30,53	3,82	2,75	11,33	18,24	-134,33	-78,80
Levofloxacin	192,74	134,04	66,45	50,89	61,13	32,97	11,33	28,66	-310,03	-69,81
Cotrimoxazol	22,68	33,51	27,69	40,71	28,68	17,86	31,73	54,09	7,77	6,24
Metronidazol	0,00	6,37	7,36	8,50	8,51	12,80	9,07	10,42	18,57	83,54
Tobramycin	9,47	9,57	0,94	11,35	18,70	0,00	0,00	0,00	-12,63	-40,31
Vancomycin	22,68	15,56	19,38	20,36	6,37	8,24	4,53	15,63	-43,21	-55,41
Linezolid	5,67	3,83	5,54	2,54	5,09	0,00	11,33	8,08	6,92	39,36
Clindamycin	1,87	0,00	14,01	0,00	6,78	0,00	6,35	6,25	3,5	22,04
Fluconazole	24,09	7,18	11,07	13,99	0,00	10,99	0,00	2,61	-42,73	-75,86
Total	822,90	715,65	646,79	723,92	645,08	545,55	535,04	640,39	-543,2	-18,67

AMS: Antimicrobial stewardship; Q: quarter.

Table S2. Recommendations for perioperative prophylaxis.

Procedure	Antibiotic	Application	Duration of Antibiotic Prophylaxis *
Transurethral resection (bladder/ prostate) or ureterorenoscopy	According to the pre-OP urine culture<br>If the culture is sterile: Ampicillin/Sulbactam **	i.v./p.o.	Relevant bacteuria: starting 2 days before OP<br>Sterile urine: single shot
Percutaneous nephrolithotomy and litholapaxy	According to the pre-OP urine culture<br>If the culture is sterile: Ampicillin/Sulbactam **	i.v./p.o.	Relevant bacteuria: starting 2 days before OP<br>Sterile urine: single shot
Prostate biopsy	Fosfomycine *	1 x 3 g p.o.<br>(1-4 h pre-OP)	Single shot
Robotic-assisted partial nephrectomy	Ampicillin/Sulbactam **	1 x 3g i.v.	Single shot
Cystectomy with ileal conduit	Ampicillin/Sulbactam **<br>Cefpodoxime *	3 x 3 g i.v. /d<br>2 x 200 mg p.o./d	3d i.v.,<br>after d4<br>total duration: 14d
Robotic-assisted radical prostatectomy	Ampicillin/Sulbactam **	1 x 3g i.v. /d	Single shot
Open nephrectomy	Ampicillin/Sulbactam **	1 x 3g i.v. /d	Single shot
Laparoscopic (partial) nephrectomy	Ampicillin/Sulbactam **	1 x 3g i.v. /d	Single shot
Retropubic radical prostatectomy	Ampicillin/Sulbactam **	3 x 3g i.v. /d	Single shot

* The preoperative dosis should be administered within 60 minutes to surgical incision; additional intraoperative dosing should be considered for surgeries lasting longer than 3 hours or in case of excessive blood loss during the procedure (> 1 liter). ** Adjust the dosis according to the kidney function.
